# Antigenic and Structural Properties of the Lipopolysaccharide of the Uropathogenic *Proteus mirabilis* Dm55 Strain Classified to a New O85 *Proteus* Serogroup

**DOI:** 10.3390/ijms242216424

**Published:** 2023-11-16

**Authors:** Agata Palusiak, Anna Turska-Szewczuk, Dominika Drzewiecka

**Affiliations:** 1 Department of Biology of Bacteria, Faculty of Biology and Environmental Protection, University of Lodz, Banacha 12/16, 90-237 Lodz, Poland; dominika.drzewiecka@biol.uni.lodz.pl; 2 Department of Genetics and Microbiology, Institute of Biological Sciences, Maria Curie-Sklodowska University, Akademicka 19, 20-033 Lublin, Poland; anna.turska-szewczuk@mail.umcs.pl

**Keywords:** *Proteus mirabilis*, lipopolysaccharide, cross-reacting LPS, O polysaccharide, NMR, antiserum, serological classification

## Abstract

The aim of the study was the serological and structural characterization of the lipopolysaccharide (LPS) O antigen from *P. mirabilis* Dm55 coming from the urine of a patient from Lodz. The Dm55 LPS was recognized in ELISA only by the O54 antiserum, suggesting a serological distinction of the Dm55 O antigen from all the 84 *Proteus* LPS serotypes described. The obtained polyclonal rabbit serum against *P. mirabilis* Dm55 reacted in ELISA and Western blotting with a few LPSs (including O54), but the reactions were weaker than those observed in the homologous system. The LPS of *P. mirabilis* Dm55 was subjected to mild acid hydrolysis, and the obtained high-molecular-mass O polysaccharide was chemically studied using sugar and methylation analyses, mass spectrometry, and ^1^H and ^13^C NMR spectroscopy, including ^1^H,^1^H NOESY, and ^1^H,^13^C HMBC experiments. The Dm55 O unit is a branched three-saccharide, and its linear fragment contains α-Gal*p*NAc and β-Gal*p*, whereas α-Glc*p*NAc occupies a terminal position. The Dm55 OPS shares a disaccharide epitope with the *Proteus* O54 antigen. Due to the structural differences of the studied O antigen from the other described *Proteus* O polysaccharides, we propose to classify the *P. mirabilis* Dm55 strain to a new *Proteus* O85 serogroup.

## 1. Introduction

The genus *Proteus*, formerly belonging to the family *Enterobacteriaceae*, has been lately recognized as a member of the newly formed family *Morganellaceae*. It has been proposed to be placed in the new family based on taxonomic changes within the order *Enterobacteriales*, renamed as *Enterobacterales* [1]. *Proteus* bacteria are Gram-negative, facultatively anaerobic, heterotrophic, proteolytic, and peritrichously flagellated motile rods [2]. The last feature accounts for the swarming phenomenon, which distinguishes *Proteus* spp. from most *Enterobacterales* [3]. The genus *Proteus* includes two species—*P. mirabilis* and *P. vulgaris*—and was first described in 1885 by a German microbiologist Gustav Hauser and now covers more than ten species. The rods may be a member of the resident microbiota in the gastrointestinal tracts of a part of the human population, and the intestines are probably a reservoir of these organisms [2,4]. At the same time, *Proteus* spp. bacteria are opportunistic human pathogens, which, in favorable conditions, may lead to cross-infections and autoinfections. Among all described *Proteus* species, *P. mirabilis* is the most frequent cause of infections [2]. It is regarded as the third most common etiological factor of urinary tract infections (UTIs), among which catheter-associated urinary tract infections (CAUTIs) occur quite frequently [5,6]. According to the National Healthcare Safety Network (NHSN, Atlanta, GA, USA) review, *Proteus* spp. account for 4% of CAUTI cases [7]. Apart from UTIs, *P. mirabilis* has been implicated in pneumonia, neonatal meningoencephalitis, empyema and osteomyelitis, and eye, ear, nose, and wound infections [3]. These bacteria are also a causative agent of diarrhea, and some studies have shown their potential role in infective endocarditis and rheumatoid arthritis development [2,8,9]. Pathogenicity of *P. mirabilis* strains is associated with numerous virulence factors such as urease, fimbriae, flagella, proteinases, and lipopolysaccharide (LPS) endotoxin, which poses a significant threat since its interaction with the host immune system may contribute to a septic shock development [3,6]. A smooth (S) LPS of *Proteus* spp. bacilli is built of three regions differing in their structures and biological functions: lipid A (structurally the most conserved region), core oligosaccharide (more structurally diverse), and O-polysaccharide (structurally the most heterogenous). Interestingly, S-form strains also possess in their outer membrane rough (R) LPS, and there are some strains that may be completely deprived of three-part LPS. It should be mentioned that S strains are considered to be more pathogenic than R forms. O polysaccharide is the most outstanding part of the bacterial cell wall and exhibits many biological activities, like getting involved in glycocalyx formation or making the bacteria resistant to the active membrane attack complex [10,11]. It is also a highly immunogenic O antigen, and its fragments (epitopes), which are common to many different bacteria, may be used as vaccine antigens inducing cross-protection in humans [10]. To date, the O antigen of *Shigella sonnei* LPS has been successfully used in vaccines administered to humans in clinical studies [12]. Thus, it is crucial to gain a better knowledge of the OPS structure and epitope specificity, which are also a basis for serological classification of the strains to an appropriate O serogroup. To date, the *Proteus* classification scheme includes 84 O serogroups and 11 R serogroups, which are still being updated and completed with new representatives [13,14]. In this work, we report on a new OPS structure and the serological specificity of the *P. mirabilis* Dm55 LPS obtained from the isolate coming from a patient from central Poland.

## 2. Results

### 2.1. Serological Studies

**Figure 1:**
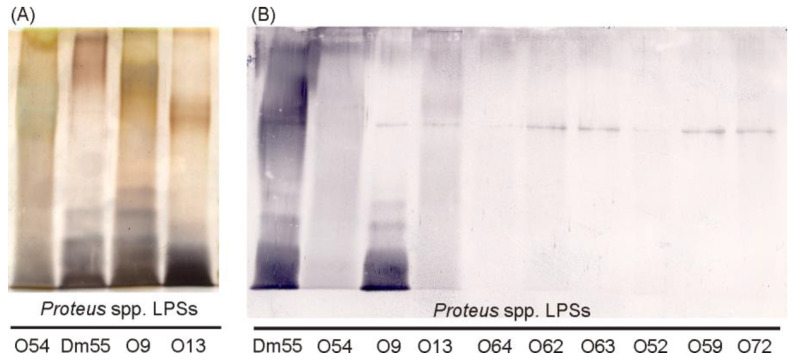
SDS-PAGE and Alcian blue–silver staining patterns of preparations strongly reacting in ELISA of the 95 tested LPSs (**A**), Western blot of *Proteus* spp. electrophoretically separated LPSs studied in reaction with *P. mirabilis* Dm55 antiserum (**B**). The LPS samples were prepared by mixing equal volumes of LPS water solution (2 mg/mL) and a loading buffer, followed by boiling for 10 min. Then, 6 μg of each LPS sample was added per lane of SDS-polyacrylamide gels and separated in SDS-PAGE.

The LPS of *P. mirabilis* Dm55 was extracted by the phenol–water method. It demonstrated a typical, ladder-like pattern in SDS-PAGE after Alcian blue–silver staining, thus proving the presence of high-molecular-mass molecules, including an O-specific long-chain polysaccharide (S form LPS) (Figure 1A).

To find out if the studied strain may be classified as one of the 84 *Proteus* O serotypes recognized so far, its LPS was tested in ELISA with the sera specific to each of the serotypes in dilutions 1:32,000 and 1:64,000. The only cross-reaction was observed with the O54 antiserum (1:64,000), but it was much weaker than in the homologous system of the O54 antiserum (1:256,000), which suggested only similarity between and not identity of both antigens (Dm55 and O54 LPSs).

At the next stage of the studies, a whole-cell vaccine containing bacterial cells of *P. mirabilis* Dm55 was prepared to obtain the polyclonal rabbit serum to confirm the suggested similarity between the Dm55 and O54 antigens. The serum reactivity was checked in ELISA with the homologous LPS as well as with the LPSs presenting each of the known 84 *Proteus* O serotypes. The *P. mirabilis* Dm55 antiserum appeared to be quite reactive since the cross-reactions were observed with many LPSs, but the serum titers were lower than in the homologous system (1:4,096,000) (Table 1).

Among the cross-reacting LPSs, five, *P. mirabilis* O54, O9, *P. vulgaris* O13, and *P. penneri* O62, O72, reacted to the titer 1:64,000 or higher. The remaining heterologous LPSs bound the antibodies with a lower intensity, and the reactivity titers of the sera specific to these LPSs were much higher (Table 1).

The reactions detected in ELISA (Table 1) were also analyzed by using the Western blotting method (Figure 1B). The homologous LPS, *P. mirabilis* Dm55, was strongly recognized by the *P. mirabilis* Dm55 serum in a way characteristic for both low- and high-migrating bands, which indicated a presence in the antiserum of both core-specific and O-polysaccharides-specific antibodies (Figure 1B). Three cross-reacting LPSs (O54, O13, and O9) were found to display in Western blotting quite intensive binding patterns, while the reactions of the remaining LPSs were less intense and concerned mainly the high-molecular-mass species containing the whole LPS molecules (Figure 1B).

The reaction patterns of low-molecular-mass species of *P. mirabilis* O9 and Dm55 LPSs were very similar in the reaction with the Dm55 antiserum, which suggests a high level of similarities in the core oligosaccharides of both LPSs. In the opposite system, the reaction of the O9 antiserum with the Dm55 LPS core region was weaker, but it was visible that the O9 antiserum was poor in anti-core antibodies (Figure S1A).

The *P. vulgaris* O13 LPS reacted in Western blotting with the *P. mirabilis* Dm55 serum at the level of both low- and high-molecular-mass species, but the intensity of its banding pattern was weaker than that of the homologous LPS, Dm55 (Figure 1B). However, in the opposite system, the O13 antiserum did not recognize the Dm55 LPS (Figure S1B).

**Figure 2:**
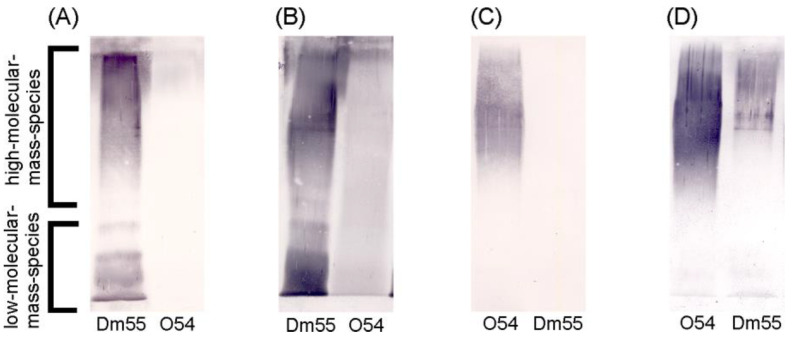
Western blotting of the *P. mirabilis* Dm55 and O54 LPSs with (**A**) the *P. mirabilis* Dm55 antiserum adsorbed with the cells of the *P. mirabilis* O54 strain, (**B**) the *P. mirabilis* Dm55 antiserum (not adsorbed), (**C**) the *P. mirabilis* O54 antiserum adsorbed with the cells of the *P. mirabilis* Dm55 strain, (**D**) the *P. mirabilis* O54 antiserum (not adsorbed).

The *P. mirabilis* O54 LPS was the one that cross-reacted with the studied Dm55 antiserum quite strongly both in ELISA and Western blotting (Table 1, Figure 1B). The cross-reaction was also observed for the opposite system, O54 antiserum with the Dm55 LPS, in the Western blot, where it was visible, especially in the region of slowly-migrating LPS molecules, rich in the OPS fractions (Figure 2D). However, the binding pattern of the Dm55 LPS was different than that of the O54 (homologous) LPS.

To confirm serological similarities between *P. mirabilis* O54 and Dm55 LPSs, the sera specific to both strains were adsorbed with the bacterial cells of the cross-reacting strain. Adsorption resulted in the weakening of the Western blotting reaction in each homologous system, which indicated some similarities but not the identity between the *P. mirabilis* Dm55 and O54 LPSs (Figure 2A–D). To see how much the reactivities of both tested sera with the homologous LPS changed after their adsorption, they were also tested in ELISA. It was demonstrated that the adsorption of the Dm55 antiserum with the cells of the *P. mirabilis* O54 strain resulted in an eightfold decrease in the reactivity titer from 4,096,000 (not adsorbed Dm55 serum) to 512,000 (adsorbed Dm55 serum). However, the reactivity of the O54 antiserum adsorbed with Dm55 biomass was reduced as much as 32-fold compared to the titer of not adsorbed O54 serum (Table 2).

Only the antibodies specific to a putative common epitope were removed from both antisera during the adsorption processes. The immunoglobulins that remained in the sera were specific to the fragments, which were different in the two OPSs (Table 2, Figure 2A,C).

The three heterologous LPSs (*P. mirabilis* O9, O54, and *P. vulgaris* O13), which strongly cross-reacted with the tested Dm55 antiserum in ELISA and Western blotting, when separated in polyacrylamide gel were also subjected to a silver staining procedure with prefixation of gel in Alcian blue. These LPSs presented different electrophoretic patterns than the Dm55 LPS (Figure 1A). The low-molecular-mass fractions of each LPS tested were silver stained with high intensity, but the most similar bands were observed for the Dm55 and O9 LPSs. What is more, these two LPSs, together with the LPS of the O13 strain, contained higher amounts of R-form molecules than the O54 LPS. As for the high-molecular-mass fractions of the LPSs, the most visible bands were obtained for the O9 and O54 LPSs, while for the O13 and Dm55 LPSs, less abundant fractions of long-chain molecules were observed (Figure 1A).

### 2.2. Structural Studies of O-Polysaccharide (OPS)

The OPS was released from the LPS of *P. mirabilis* Dm55 by mild-acid degradation followed by centrifugation of the lipid A precipitate and isolated, in a void volume, by gel-permeation-chromatography (GPC) on a Sephadex G50 fine column. The yield of the high-molecular-mass OPS fraction was 15% of the LPS mass subjected to hydrolysis. The GLC-MS sugar analysis of alditol acetates obtained after full acid hydrolysis of the OPS with 2 M CF_3_CO_2_H showed the presence of galactose (Gal), glucosamine (GlcN), and galactosamine (GalN) as the major components, in a peak area ratio of 1.0:1.0:1.4. The alditol acetates GLC-MS data have been shown in Supplementary Figure S2.

The determination of the absolute configuration of the monosaccharides by GLC of acetylated (*S*)-2-octyl glycosides indicated the presence of d-Gal, d-GlcN, and d-GalN.

The methylation analysis of the OPS completed the compositional data and resulted in identification of 1,3,5,6-tetra-*O*-acetyl-2,4-di-*O*-methylhexitol-1-*d* (derived from 3,6-disubstituted Gal), 1,5-di-*O*-acetyl-2-deoxy-3,4,6-tri-*O*-methyl-2-(*N*-methyl)acetamidohexitol-1-*d* (derived from terminal GlcN) and 1,3,5-tri-*O*-acetyl-2-deoxy-4,6-di-*O*-methyl-2-(*N*-methyl)acetamidohexitol-1-*d* (derived from 3-substituted GalN), with a peak area ratio of 1.0:1.2:1.3, identified by GLC-MS. The permethylated alditol acetates GLC-MS data have been shown in Supplementary Figure S3.

The O-polysaccharide structure of *P. mirabilis* Dm55 was then studied with the use of 1D and 2D NMR spectroscopy.

**Figure 3:**
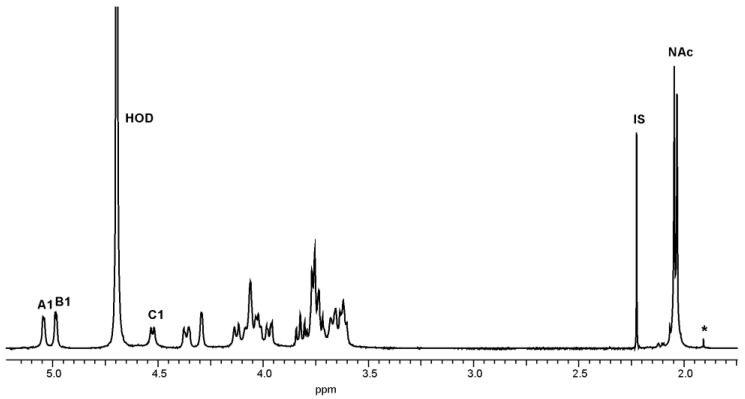
The ^1^H NMR spectrum of the OPS of *P. mirabilis* strain Dm55. The spectrum was recorded at 32 °C in D_2_O at 500 MHz. The capital letters and Arabic numerals refer to the atoms in the sugar residues denoted, as shown in Table 3. NAc, *N*-acetyl groups, IS—acetone as an internal standard (*δ* 2.225), asterisk—free acetic acid.

The ^1^H NMR spectrum of the O-polysaccharide (Figure 3) showed three signals for anomeric protons at *δ* 5.04, 4.99, and 4.53, with an integral intensity ratio of 1.0:1.04:0.98, indicating that the O-polysaccharide had a regular structure. There were also two signals for *N*-acetyl groups at *δ* 2.04 and the ring proton signals in the range of δ 3.62–4.37, some of which overlapped.

The analysis of the two-dimensional homonuclear (^1^H,^1^H DQF-COSY, TOCSY, and NOESY) and heteronuclear (^1^H,^13^C HSQC, ^1^H,^13^C H2BC, and ^1^H,^13^C HMBC) NMR experiments resulted in the assignment of the ^1^H and ^13^C resonances to the OPS of *P. mirabilis* Dm55. The ^1^H and ^13^C NMR chemical shifts are presented in Table 3.

**Figure 4:**
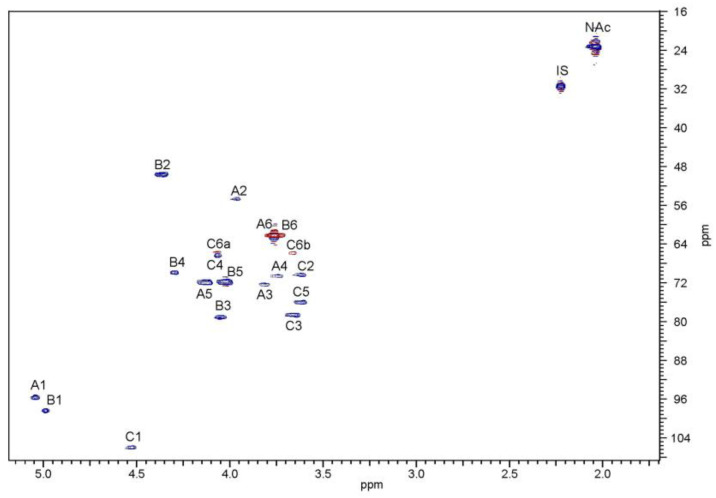
^1^H,^13^C HSQC spectrum (500 × 125 MHz) of the OPS of *P. mirabilis* strain Dm55. The spectrum was recorded at 32 °C in D_2_O as a solvent. The capital letters and Arabic numerals refer to proton/carbon pairs in the respective sugar residues denoted as follows: **A**, Glc*p*NAc; **B**, Gal*p*NAc; **C**, Gal*p*; IS—acetone as an internal standard (*δ*_H_ 2.225, *δ*_C_ 31.45).

The ^1^H,^13^C HSQC spectrum (Figure 4) contained two correlation signals at *δ* 3.97/54.7 and 4.36/49.7 of protons at the nitrogen-bearing carbons to the corresponding carbons and showed that the OPS repeating unit contained *N*-acetamido sugars. Moreover, the absence of signals at the ^13^C coordinate in the region of *δ* 83.0–88.0 characteristics of C-4 furanoses and the anomeric carbons, which should have had higher chemical shift values due to the less shielded nuclei than in their corresponding counterparts, demonstrated that all the sugars were pyranoses [15].

The ^1^H,^1^H TOCSY and DQF-COSY spectra revealed three spin systems for monosaccharides, which were labeled **A**, **B**, and **C** in the order of the decreasing chemical shifts of their H-1 protons. In the ^1^H,^1^H DQF COSY and TOCSY spectra, correlations of H-1/H-2 up to H-6 typical of monosaccharide having the *gluco* configuration were found for spin system **A**. Then, the carbon resonances were inferred from the ^1^H,^13^C HSQC spectrum, which also showed the correlations of H-2 at *δ* 3.97 with the nitrogen-bearing carbon at *δ* 54.7, and H-6 protons with C-6 at *δ* 3.76/62.2. Based on these data and after including ^3^*J*_H1,H2_ and ^1^*J*_C1,H1_ coupling constant values of ~3.3 Hz and 174 Hz, respectively, spin system **A** was assigned to α-GlcN [16].

In the ^1^H,^1^H TOCSY spectrum, starting from the H-1 proton, the correlations with H-2, H-3, and H-4 were visible for spin systems **B** and **C**, indicating monosaccharides with *galacto* configuration. The remaining resonances were assigned from the NOESY, DQF-COSY, and heteronuclear experiments.

**Figure 5:**
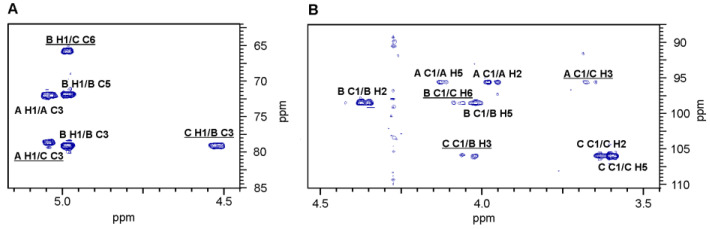
Regions of the ^1^H,^13^C HMBC spectrum of the OPS of *P. mirabilis* strain Dm55. The long-range heteronuclear correlations for (**A**) anomeric protons and (**B**) anomeric carbons are marked. Interresidue correlations important for establishing the sugar sequence in the O-antigen repeating unit are underlined. The capital letters and Arabic numerals refer to protons or carbons in the sugars denoted, as shown in Table 3.

In the ^1^H,^13^C HMBC spectrum (Figure 5), correlations of the anomeric proton at *δ* 4.99 with carbons C-3 and C-5 at *δ* 79.2 and 71.8, respectively, were found for spin system **B**, and then the proton resonances were assigned from the ^1^H,^13^C HSQC spectrum.

Moreover, in this spectrum (Figure 4), the correlation signal at *δ* 4.36/49.7 (H-2/C-2) of the proton at the nitrogen-bearing carbon to the corresponding carbon was assigned to spin system **B**. In the NOESY spectrum, correlations of H-4/H-5 and H-4/H-6 at *δ* 4.29/4.03 and 4.29/3.76, respectively, were visible for this residue. By including the ^3^*J*_H1,H2_ and ^1^*J*_C1,H1_ coupling constant values of 3.3 Hz and 174 Hz, respectively, spin system **B** was assigned to α-GalN [16].

**Figure 6:**
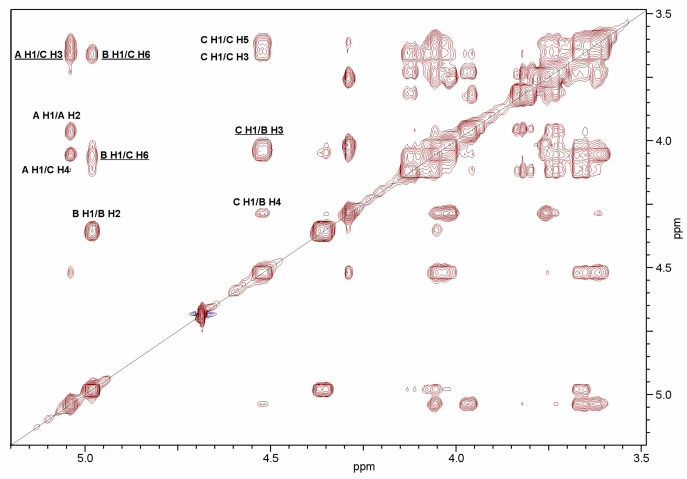
Part of the ^1^H,^1^H NOESY spectrum of the OPS of *P. mirabilis* strain Dm55. The NOE correlations between anomeric protons and protons at the glycosidic linkages are underlined. Some other important H/H correlations are depicted as well. The capital letters and Arabic numerals refer to protons in the sugar residues denoted as shown in Table 3.

On the other hand, for spin system **C**, in the NOESY spectrum (Figure 6), the *intra*-residue correlations of H-1/H-3 and H-1/H-5 characteristics of β-configurated sugars were found, and then the H-3/H-5, H-4/H-5, and H-4/H-6 correlations were analyzed.

However, given the coincidence of the H-2/H-3 and H-3/H-5 as well as H-4/H-5 and H-4/H-6 correlation signals, the chemical shifts of corresponding C-2-C-6 carbons, which were inferred from the ^1^H,^13^C HSQC spectrum, were assigned after consideration of the two-bond and long-range correlations in the ^1^H,^13^C H2BC and ^1^H,^13^C HMBC spectra, methylation analysis data and the glycosylation effects on the ^13^C NMR resonances [17]. The ^3^*J*_H1,H2_ and ^1^*J*_C1,H1_ coupling constant values of 7.7 Hz and 165 Hz, respectively, confirmed that spin system **C** was β-Gal [16].

The ^13^C resonance of the NAc carbonyl signals was assigned from the correlations between H-2 of residues **A** and **B** (*δ* 3.97 and 4.36) and the corresponding carbons in the HMBC spectrum (*δ* 175.6) and between the latter and the methyl proton signals at *δ* 2.04, respectively.

The NOESY spectrum (Figure 6) showed *intra*-residue cross-peaks: H-1/H-2 for α-Glc*p*NAc and α-GalNAc (**A** and **B**) and H-1/H-3, and H-1/H-5 for β-Gal*p* **C**, which confirmed the anomeric configurations of the sugar residues [18].

The low-field displacement of the carbon atoms C-3 (*δ* 78.7) and C-6 (*δ* 65.8) of spin system **C** and C-3 of the **B** residue (*δ* 79.2), compared with their resonances in the corresponding non-substituted monosaccharides [17], unlike the C-2,3,4,6 carbon atoms of spin system **A** showing the insignificant displacements, confirmed the glycosylation pattern of the monosaccharides in the oligosaccharide unit [19]. These data demonstrated that the O-antigen repeating unit is branched with residue **C** at the branching point and a terminal position of residue **A**.

The sugar sequence in the OPS repeating unit was determined in the homo- and heteronuclear experiments. The ^1^H,^1^H NOESY spectrum (Figure 6) showed correlation signals for pairs of transglycosidic protons, i.e., **B** H-1/**C** H-6, **C** H-1/**B** H-3, **A** H-1/**C** H-3 at *δ* 4.99/3.66;4.06, 4.53/4.04, and 5.04/3.66, respectively, thus indicating a sequence **B** → **C** → **B** in the linear part of the O-unit, and the α-Glc*p*NAc residue (**A**) located at the terminal position and glycosylating the β-Gal*p* residue (**C**) at position C-3. Moreover, in the spectrum, the additional *inter*-residue NOE contacts for **C** H-1/**B** H-4 at *δ* 4.53/4.29 and **A** H-1/**C** H-4 at *δ* 5.04/4.06 were observed, typical of (1 → 3)-linked sugars, which confirmed the glycosylation of α-Glc*p*NAc (**B**) and β-Gal*p* (**C**) at carbon C-3 [18]. Full range of ^1^H,^1^H NOESY spectrum of the OPS of *P. mirabilis* strain Dm55 has been shown in Supplementary Figure S4.

In the ^1^H,^13^C HMBC spectrum (Figure 5), the following correlations between anomeric protons and transglycosidic carbons were observed: Gal*p*NAc **B** H-1/ Gal*p* **C** C-6 at *δ* 4.99/65.9, Gal*p* **C** H-1/ Gal*p*NAc **B** C-3 at *δ* 4.53/79.2, and Glc*p*NAc **A** H-1/ Gal*p* **C** C-3 at *δ* 5.04/78.7. Furthermore, the following correlations between anomeric carbons and transglycosidic protons were also visible in the spectrum: Gal*p*NAc **B** C-1/ Gal*p* **C** H-6 at *δ* 98.4/4.06, Gal*p* **C** C-1/ Gal*p*NAc **B** H-3 at *δ* 105.9/4.04, and Glc*p*NAc **A** C-1/ Gal*p* **C** H-3 at *δ* 95.6/3.66. Full range of ^1^H,^13^C HMBC spectrum of the OPS of *P. mirabilis* strain Dm55 has been shown in Supplementary Figure S5.

**Scheme 1:**
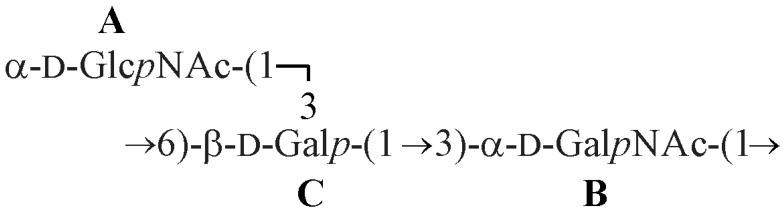
Structure of the OPS repeating unit of *P. mirabilis* Dm55.

In conclusion, the data showed that the O-specific polysaccharide from *P. mirabilis* Dm55 had the structure presented below (Scheme 1).

## 3. Discussion

The aim of the study was the serological and structural characterization of the LPS O antigen from the strain *P. mirabilis* Dm55 coming from the urine of a patient from Lodz (Poland). To date, such characterization has been performed for ten *Proteus* spp. strains infecting patients from central Poland and isolated from different sources, among which four isolates were found as uropathogenic *P. mirabilis* [13,14,20]. Urine was the most common source (43%) of *Proteus* spp. isolates among 617 clinical strains coming from that area, and *P. mirabilis* appeared to be the predominant species (90.3%). Most of these strains have already been classified to numerous O serogroups, and the O serotypes most prevalent in central Poland have been indicated [13].

The scenario of the serological studies in this work was realized using reliable and complementary techniques successfully employed in our previous research and also in other studies (ELISA as a highly sensitive serological method and Western blotting due to its specificity).

The first stage of the serological classification of the *P. mirabilis* Dm55 LPS was an analysis of its similarity to *Proteus* LPS serotypes described so far by testing its specificity with the O1–O84 *Proteus* antisera in ELISA. A lack of strong cross-reactions with the set of the tested antisera observed for the Dm55 LPS suggested its serological distinction from all known *Proteus* O serotypes. Thus, in the next stage, a polyclonal rabbit antiserum was obtained for the studied Dm55 strain, which was rich in O-specific antibodies, but also anti-core antibodies were present in large amounts (Figure 1B). The situation is not rare, and such antiserum may be applied to the determination of similarities not only in O antigens but also in the LPS core region in various strains [13]. In ELISA, the *P. mirabilis* Dm55 antiserum strongly cross-reacted with a few *Proteus* spp. LPS (Table 1). However, in Western blotting (Figure 1B), cross-reactions with some LPSs (O52, O59, O62-O64, O72) were barely visible. Due to the lower specificity and, simultaneously, higher sensitivity of reactions in ELISA compared to Western blotting [21], the less specific cross-reactions were not confirmed by the latter method.

The LPSs for which the most intensive banding patterns were obtained in Western blotting with the *P. mirabilis* Dm55 antiserum were subjected to a silver staining procedure with prefixation of gel in Alcian blue after SDS-PAGE. Such a combination of staining methods allows detection of even very small amounts of acidic polysaccharides, components typical for *Proteus* spp. O-polysaccharides of LPSs, which makes the procedure more sensitive than silver staining without a previous gel fixation with Alcian blue [22]. The electrophoregram of LPS preparations from the strains Dm55, O9, O13, and O54 (Figure 1A) shows different staining patterns of separated LPS molecules varying in the numbers of the repeating units. The tested Dm55 LPS was less intensively stained within the molecules consisting of O-chains compared to the O9 and O54 LPSs. However, the studied Dm55 OPS does not contain cationic groups; thus, it may have been less clearly stained than the remaining ones containing some acidic components [11], which are the additional binding sites for the anionic dye Alcian blue (Figure 1A).

**Figure 7:**
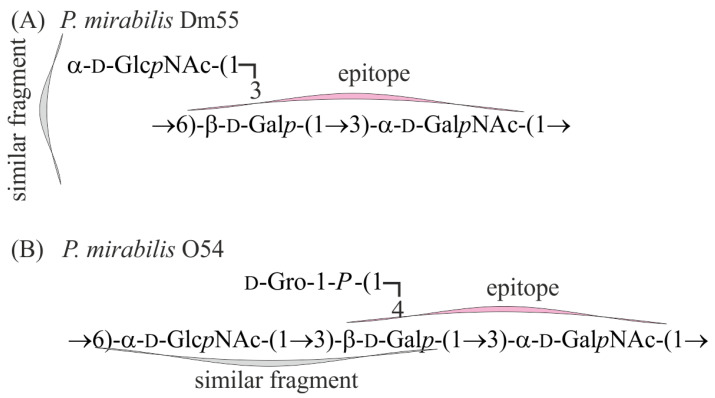
Structures of the O repeating unit of *P. mirabilis* Dm55 (**A**) and O54 [11]. (**B**) LPSs The common epitope and the similar fragment are indicated by arcs. d-Gal, d-galactose; d-GalNAc, 2-acetamido-2-deoxy-d-galactose (*N*-acetyl-d-galactosamine); d-GlcNAc, *N*-acetyl-d-glucosamine; d-Gro-1-*P,* d-Glycerol 1-phosphate.

As was mentioned before, the only serum that cross-reacted with the *P. mirabilis* Dm55 LPS in ELISA was that specific to the *P. mirabilis* O54 strain (the first stage of the studies). This fact and quite strong serological activity observed for the opposite system (*P. mirabilis* Dm55 antiserum and O54 LPS) in ELISA and Western blotting suggested that these two LPSs, *P. mirabilis* Dm55 and O54, share some high structural similarities (Table 1, Figure 1B). The chemical and structural analyses of the *P. mirabilis* Dm55 OPS conducted in this work confirmed this hypothesis. Both Dm55 and O54 LPSs (this work, [11]) possess in their OPS repeating units two following common fragments: β-d-Gal*p*-(1 → 3)-α-d-Gal*p*NAc, which may play a role of a common epitope and α-d-Glc*p*NAc-(1 → 3)-β-d-Gal*p* (indicated as a similar fragment in Figure 7).

The latter fragment in the studied Dm55 OPS is side-branched, while in *P. mirabilis* O54 OPS, it is part of a linear structure of its repeating unit. What is more, in O54 OPS, the d-β-Gal*p* residue is additionally substituted with the d-Gro-1-*P* residue, which is absent in Dm55 OPS (Figure 7). Thus, despite the similar composition of the two OPSs, the structural differences between them strongly influenced their serological specificity and reactivity of both studied antisera in the cross-reactions (the lower reciprocal titers in ELISA and weaker electrophoretic bands in Western blots when compared to those observed for homologous systems—Table 1, Figure 1B and Figure 2D. The differences between the O antigens accounted for a slight decrease in the sera reactivity in the homologous systems after their adsorption with the cells of the cross-reacting strains (Table 2).

O13 and O9 LPSs were recognized by the Dm55 antiserum (Figure 1B), but the reactions in the opposite systems were hardly visible (Figure S1A,B). Moreover, no common structural fragments were found in their OPSs and Dm55 O antigen [11]. The data indicate that, most probably, only the core-specific antibodies contributed to the observed one-sided cross-reactions of the Dm55 antiserum (Figure 1B).

## 4. Materials and Methods

### 4.1. The Studied Strains, Lipopolysaccharides (LPSs), and Polyclonal O-Specific Sera

The studied strain, *P. mirabilis* Dm55, was isolated in 2009 from the urine of a 35-year-old man and was kindly provided by the Diagnostyka Laboratory in Lodz, Poland. According to Senior [23], the analyzed biochemical properties of the strain confirm that it belongs to the *P. mirabilis* species (positive in phenyloalanine, ornithine, and urea utilization, while tryptophane, mannose, and salicin—negative). The strain was stored at −80 °C and cultivated using a nutrient broth medium.

The LPS of the strain was obtained by applying the classical phenol–water method [24]. Briefly, the LPS was extracted from the lyophilized biomass of the strain using 45% phenol at 65 °C for 30 min. After the removal of the biomass residues (centrifugation) and phenol (dialysis to water), the LPS solution was concentrated and purified (to remove proteins and nucleic acids) using 2.5 M trichloroacetic acid (TCA) to achieve pH 2. The precipitated contaminations were removed by centrifugation (5285× *g*), and TCA was dialyzed to water. Then, the LPS sample was lyophilized.

The Dm55 antiserum was obtained according to a described procedure [25] via an 18-day vaccination of a White New Zealand rabbit by applying three doses of the 1.5 × 10^10^ suspension of heat-killed bacterial cells, with the approval of the local ethical committee from 17 July 2006 (The Ninth Local Ethical Committee on Animal Testing in Lodz, permission number 29/ŁB333/2006).

LPS samples representing O1–O84 *Proteus* serotypes, as well as the O1–O84-specific sera, belong to the Department of Biology of Bacteria.

Each selected antiserum was adsorbed three times using a wet biomass of a particular cross-reacting strain in a volume ratio of 1:10. Lack of reaction of an adsorbed serum with the homologous LPS confirmed that the adsorption was proper and complete [20].

### 4.2. Enzyme-Linked Immunosorbent Assay (ELISA)

ELISA was performed according to the method by Drzewiecka et al. [13], with some modifications. Flat-bottom 96-well-titrate plates were used, and the wells were coated with 50 ng of the studied LPS in 50 µL phosphate saline buffer. The analyzed antisera were serially diluted (q = 2), which allowed for determining the titer—the last dilution when the reaction was considered to be positive. The reactions were visualized using rabbit-IgG specific peroxidase-conjugated goat antibodies (Jackson ImmunoResearch, West Grove, PA, USA) and 2,2′-Azino-bis(3-ethylbenzothiazoline-6-sulfonic acid) diammonium salt (ABTS) (Sigma-Aldrich, St. Louis, MO, USA) as a substrate for peroxidase. The absorbance (A_405_) was measured with the use of a Multiskan Go microplate reader (Thermo Fisher Scientific, Vantaa, Finland).

### 4.3. Polyacrylamide Gel Electrophoresis (PAGE) of LPS and Western Blotting

The LPS samples were prepared by mixing equal volumes of LPS water solution (2 mg/mL) and a loading buffer (2% SDS and 50 mM Tris/HCl (pH 6.8), 25% glycerol, 0.1% bromophenol blue), followed by boiling for 10 min. Then, 6 μg of each LPS sample was added per lane of SDS-polyacrylamide gels and separated in the electric field (200 V). When separated, LPS bands were visualized in gels by staining with silver nitrate and 0.01% Alcian Blue [22,26] or transferred to nitrocellulose membranes in the electric field (100 V). Then, the serological reactions with analyzed O-antisera observed in ELISA were visualized, applying goat anti-rabbit-IgG antibodies conjugated with alkaline phosphatase (AP) (Jackson ImmunoResearch, West Grove, PA, USA) and a proper AP Conjugate Substrate Kit (Bio-Rad, Hercules, CA, USA) [13].

### 4.4. Degradation of LPS and Isolation of O-Polysaccharide

The LPS sample (120 mg) of *P. mirabilis* Dm55 was heated in 2% acetic acid at 100 °C for 3 h, and the lipid A precipitate was removed by centrifugation (12,000× *g*, 30 min). The supernatant was concentrated and then fractionated by gel-permeation chromatography (GPC) on a column (1.8 cm × 80 cm) of Sephadex G-50 fine (Pharmacia, Uppsala, Sweden) using 1% acetic acid as an eluent and monitoring with a differential refractometer (Knauer, Berlin, Germany).

### 4.5. Chemical Analyses

For neutral and amino sugar analysis, the OPS sample of *P. mirabilis* Dm55 was hydrolyzed with 2 M CF_3_CO_2_H (120 °C, 2 h), reduced with NaBD_4_, and peracetylated with a 1:1 (*v*/*v*) Ac_2_O-pyridine mixture (85 °C, 0.5 h). Alditol and aminoalditol acetate derivatives were analyzed by GLC-MS.

The absolute configuration of monosaccharides was determined by GLC of acetylated (*S*)-(+)-2-octyl- and racemic 2-octyl-glycoside derivatives using authentic sugar standards as described previously [27].

Methylation analysis of the OPS (1.5 mg) was carried out with methyl iodide in dimethyl sulfoxide in the presence of powdered sodium hydroxide as described elsewhere [28]. The products were recovered by extraction with chloroform/water (1:1, *v*/*v*), *N*-acetylated, hydrolyzed with 2 M CF_3_CO_2_H (120 °C, 2 h), *N*-acetylated, reduced with NaBD_4_ and peracetylated. The partially methylated alditol and aminoalditol acetates (PMAA) were analyzed by GLC-MS.

All the sugar derivatives were analyzed on an Agilent Technologies 7890A gas chromatograph (Wilmington, DE, USA) connected to a 5975C MSD detector (inert XL EI/CI, Agilent Technologies, Wilmington, DE, USA). The chromatograph was equipped with an HP-5MS capillary column (Agilent Technologies, 30 m × 0.25 mm, flow rate of 1 mL min^−1^, He as a carrier gas). The temperature program was as follows: 150 °C for 5 min, then 150 to 310 °C at 5 °C min^−1^, and the final temperature was maintained for 10 min.

### 4.6. NMR Spectroscopy

The OPS sample (7 mg) was deuterium-exchanged by freeze-drying from a 99.95% D_2_O solution and examined in 99.98% D_2_O. 1D and 2D NMR spectra were recorded at 32 °C on a 500 MHz NMR Varian Unity Inova instrument (Varian Associates, Palo Alto, CA, USA) and calibrated with internal acetone (*δ*_H_ 2.225, *δ*_C_ 31.45). Standard Varian software Vnmrj V. 4.2 rev. (Agilent Technologies, Santa Clara, CA, USA) was used to acquire and process the NMR data. Homonuclear and heteronuclear two-dimensional experiments: ^1^H,^1^H TOCSY, ^1^H,^1^H DQF-COSY, ^1^H,^1^H NOESY, ^1^H,^13^C HSQC, ^1^H,^13^C H2BC, and ^1^H,^13^C HMBC were conducted for signal assignments and determination of the sugar sequence in the repeating unit. The mixing time of 100 and 200 ms was used in the TOCSY and NOESY experiments, respectively. The ^1^H,^13^C HSQC spectrum (band-selective gHSQCAD) measured without ^13^C decoupling was used to determine the ^1^*J*_C1,H1_ coupling constants for the anomeric carbons. The heteronuclear multiple-bond correlation (^1^H,^13^C HMBC) experiment was optimized for *J*_C,H_ = 8 Hz, with a 2-step low-pass filter of 130 and 165 Hz to suppress one-bond correlations.

## 5. Conclusions

The serological and structural results presented in this work proved the uniqueness of the *P. mirabilis* Dm55 O antigen, which is different from the 84 *Proteus* O serotypes described. These data encouraged us to create a new subsequent *Proteus* O85 serogroup, with the *P. mirabilis* Dm55 strain as its representative. So far, the *Proteus* spp. isolates from the patients living in central Poland have been classified into seven newly formed *Proteus* O serogroups, O77-O82 and O84, among which group O78 appeared to be the most numerous [13,14]. The uniqueness of the OPS structure of the *P. mirabilis* Dm55 LPS also concerns the number of components in the O-repeating unit since a branched three-saccharide unit has been found so far only in two *Proteus* O serotypes (O6 and O13) [11].

The serological and structural data presented in this paper show how important the particular configuration and position of OPS components are in the LPS specificity. Even small changes in the LPS structure may contribute to a better adaptation of bacteria to the host environment by avoiding being captured by specific antibodies [29]. The changeability and variety of clinical strains may be important properties, increasing their chances of evasion. The large number of the O serotypes found among the *Proteus* clinical isolates in central Poland, including the new ones that have been recently described, confirms the observation.

## Figures and Tables

**Table 1:** Reactivity in ELISA of Dm55 antiserum with the most reactive LPSs studied compared to the homologous reactions of these LPSs. The raw data (absorbance values) obtained for the observed reactions can be found in the Supplementary Materials Tables S1 and S2.

*Proteus* spp. LPSs	Reciprocal Titre of:	
	Dm55 Antiserum with the Cross-Reacting LPSs	Homologous Antisera
Dm55	-	4,096,000
O54	64,000	256,000
O9	128,000	256,000
O13	512,000	512,000
O64	32,000	2,048,000
O62	64,000	>4,096,000
O63	16,000	512,000
O52	16,000	2,048,000
O59	8000/16,000	128,000
O72	64,000	128,000

**Table 2:** The reactivity of intact and adsorbed *P. mirabilis* Dm55 and O54 antisera with the studied LPSs in ELISA. The data obtained for each homologous system, after and without adsorption procedure, are in bold.

	Reciprocal Titer in the Reaction with	
***P. mirabilis* Dm55 Antiserum**	**Dm55 LPS**	**O54 LPS**
not adsorbed	**4,096,000**	64,000
adsorbed with *P. mirabilis* O54 biomass	**512,000**	<2000
***P. mirabilis* O54 Antiserum**		
not adsorbed	64,000	**256,000**
adsorbed with *P. mirabilis* Dm55 biomass	<2000	**8000**

**Table 3:** ^1^H (500 MHz) and ^13^C NMR (125 MHz) data (δ, ppm) for the OPS of *P. mirabilis* Dm55.

Sugar Residue		H-1 C-1	H-2 C-2	H-3 C-3	H-4 C-4	H-5 C-5	H-6 C-6	NAc
α-d-Glc*p*NAc	**A**	5.04	3.97	3.82	3.74	4.13	3.76	2.04
		95.6	54.7	72.2	70.6	71.9	62.2	23.3; 175.6
→3)-α-d-Gal*p*NAc	**B**	4.99	4.36	4.04	4.29	4.03	3.76	2.04
		98.4	49.7	79.2	69.8	71.8	62.2	23.3; 175.6
→3,6)-β-d-Gal*p*	**C**	4.53	3.62	3.66	4.06	3.62	3.66; 4.06	
		105.9	70.3	78.7	66.4	76.0	65.8	

**A**, **B**, **C**—the note sugar residues; the glycosylated carbon atoms are underlined.

## Data Availability

The data that support the findings of this study are available from the corresponding author on reasonable request.

## Supplementary Materials

The following supporting information can be downloaded at: https://www.mdpi.com/article/10.3390/ijms242216424/s1.
