# Supplementary material for: Antigenic and Structural Properties of the Lipopolysaccharide of the Uropathogenic Proteus mirabilis Dm55 Strain Classified to a New O85 Proteus Serogroup
Source: Int J Mol Sci. 2023 Nov 16;24(22):16424. doi: 10.3390/ijms242216424 (PMC10671486; doi:10.3390/ijms242216424)
Supplement: Supplementary file 1 [file ijms-24-16424-s001.zip › Supplementary Figure S1.pdf]

Figure S1.

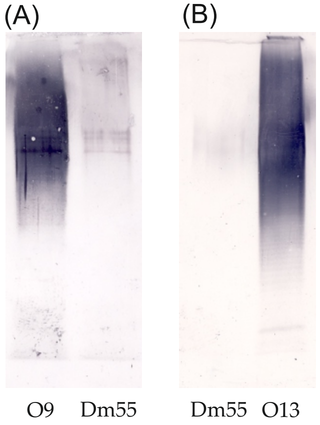

Western blotting of *Proteus* spp. electrophoretically separated lipopolysaccharides studied in the reactions with: the *P. mirabilis* O9 antiserum (A), and the *P. vulgaris* O13 antiserum (B).
