# Supplementary material for: Antigenic and Structural Properties of the Lipopolysaccharide of the Uropathogenic Proteus mirabilis Dm55 Strain Classified to a New O85 Proteus Serogroup
Source: Int J Mol Sci. 2023 Nov 16;24(22):16424. doi: 10.3390/ijms242216424 (PMC10671486; doi:10.3390/ijms242216424)
Supplement: Supplementary file 1 [file ijms-24-16424-s001.zip › Supplementary Figures S2 and S3.pdf]

Supplementary Materials:

Figure S2. The alditol acetates GLC-MS data (**A-D**) of the OPS of *P. mirabilis* strain Dm55. (**A**) The alditol acetates GLC of the OPS of *P. mirabilis* strain Dm55. (**B**) The electron impact (*EI*) mass spectrum of peracetylated hexitol(Gal)-1-*d*. (**C**) The mass spectrum (*EI*) of peracetylated 2-acetamido-hexitol(Glc)-1-*d*. (**D**) The mass spectrum (*EI*) of peracetylated 2-acetamido-hexitol(Gal)-1-*d*.

A

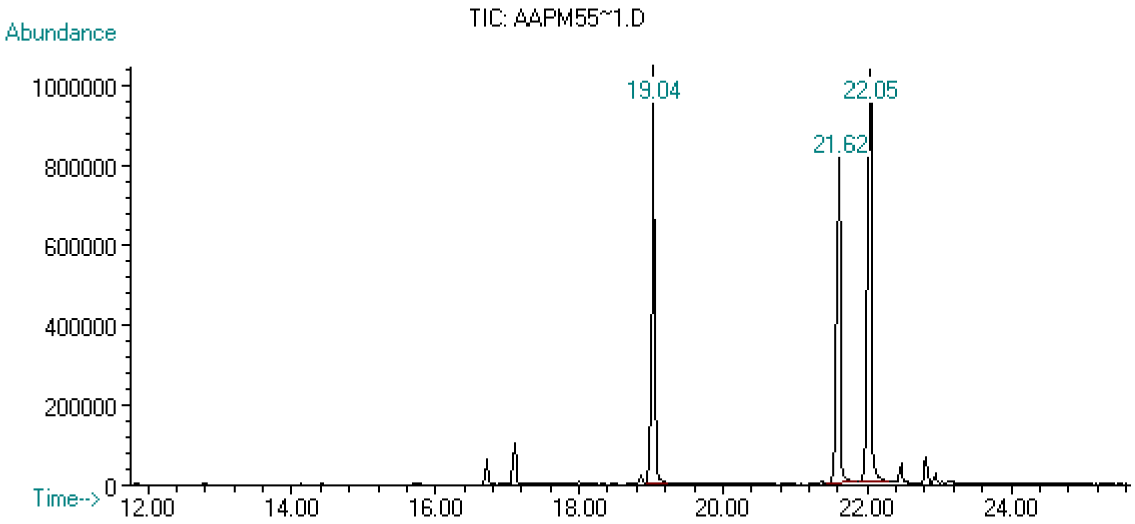

B

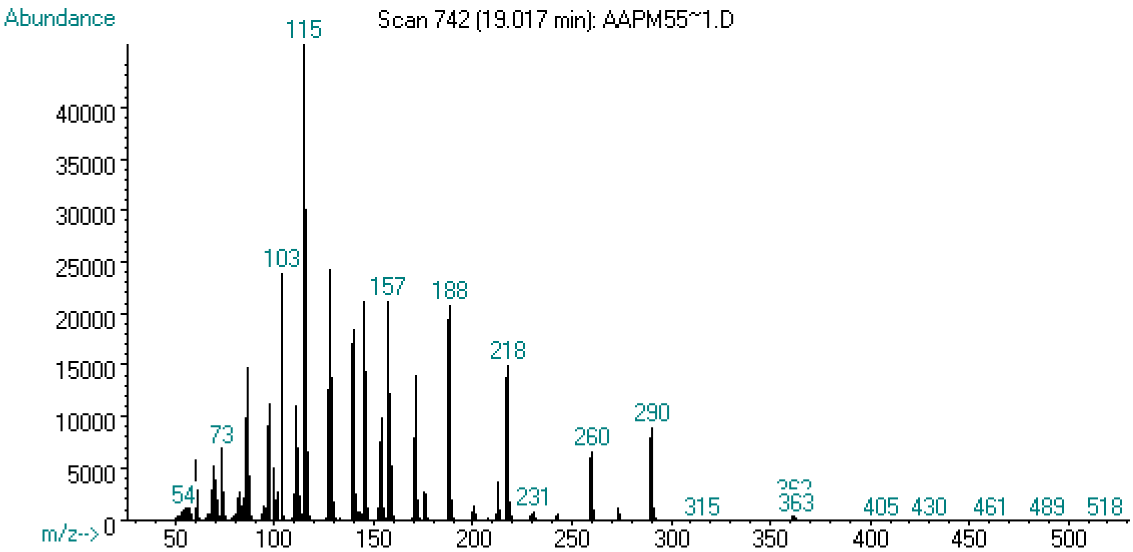

C

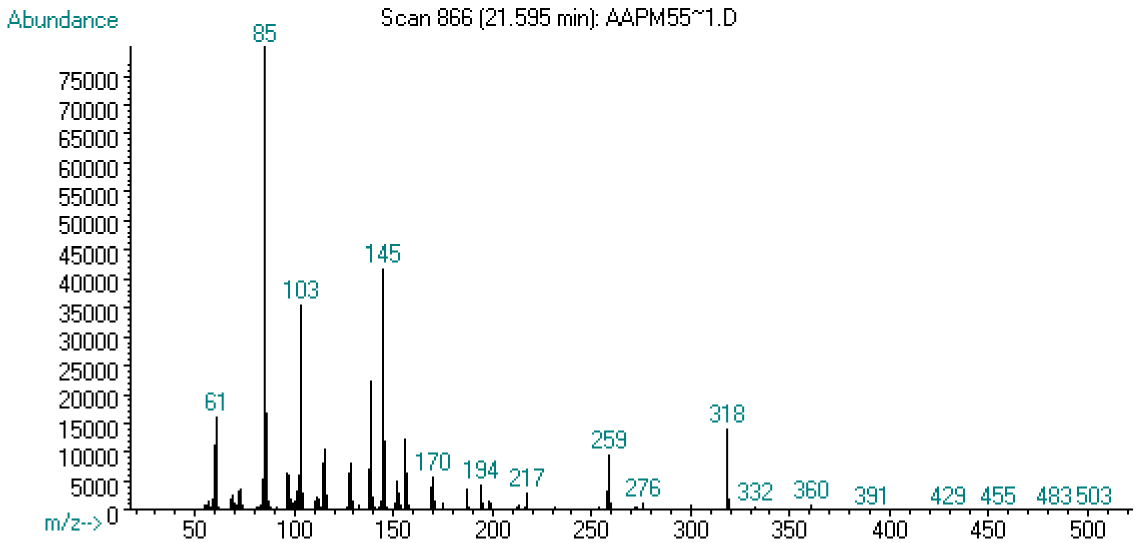
Abundance
Scan 866 (21.595 min): AAPM55~1.D
85
145
103
61
318
259
170
194
217
276
332
360
391
429
455
483
503
75000
70000
65000
60000
55000
50000
45000
40000
35000
30000
25000
20000
15000
10000
5000
0
m/z-->
50
100
150
200
250
300
350
400
450
500

D

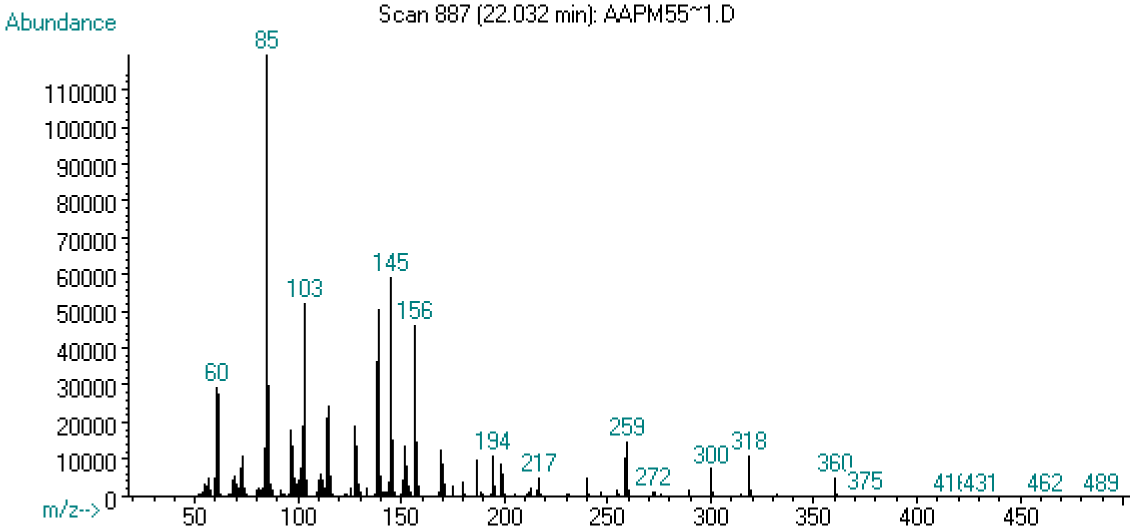
Abundance
Scan 887 (22.032 min): AAPM55~1.D
85
145
103
156
60
259
194
217
272
300
318
360
375
41(431
462
489
110000
100000
90000
80000
70000
60000
50000
40000
30000
20000
10000
0
m/z-->
50
100
150
200
250
300
350
400
450

Figure S3. The permethylated alditol acetates (PMAA) GLC-MS data (**A**-**D**) of the OPS of *P. mirabilis* strain Dm55. (**A**) Permethylated alditol acetates GLC of the OPS of *P. mirabilis* strain Dm55. (**B**) The mass spectrum (*EI*) of peracetylated 2,4-di-OMe-Hex(Gal)-1-*d*. (**C**) The mass spectrum (*EI*) of peracetylated 3,4,6-OMe-2-NMeAc-HexN(GlcN)-1-*d*. (**D**) The mass spectrum (*EI*) of peracetylated 4,6-OMe-2-NMeAc-HexN(GalN)-1-*d*.

A

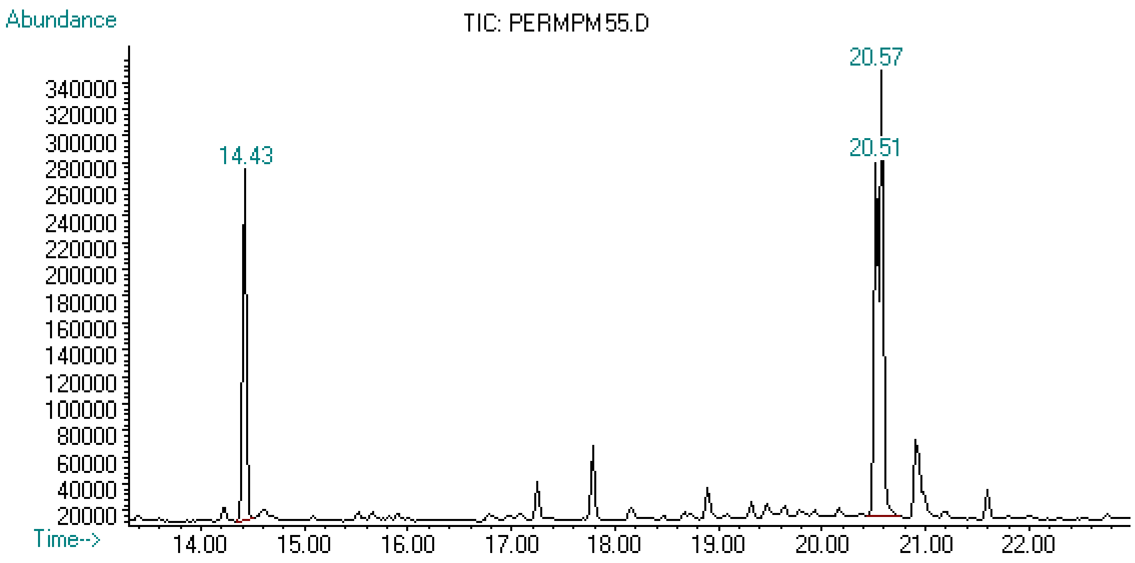

B

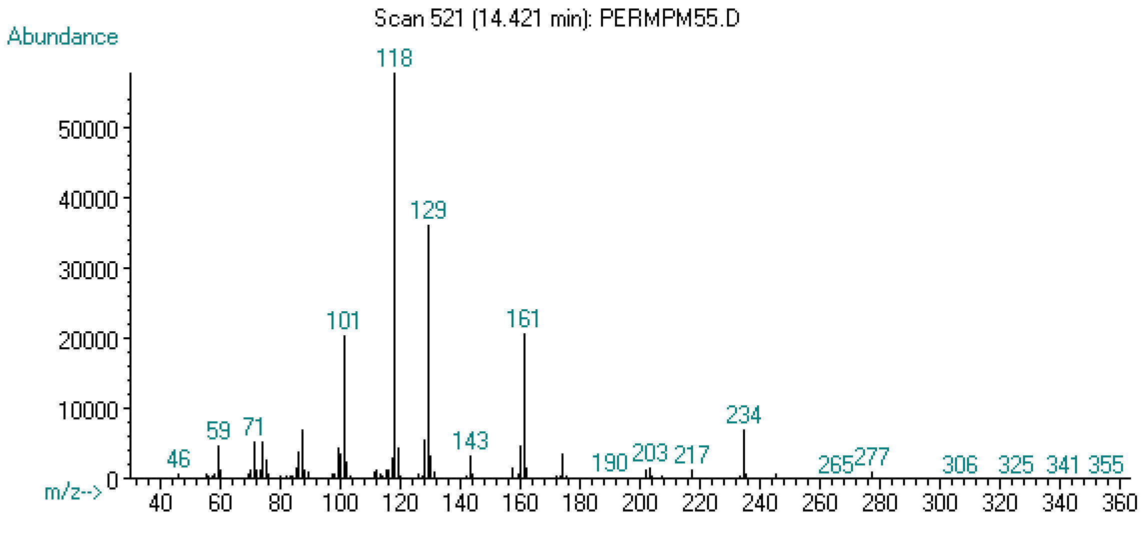

C

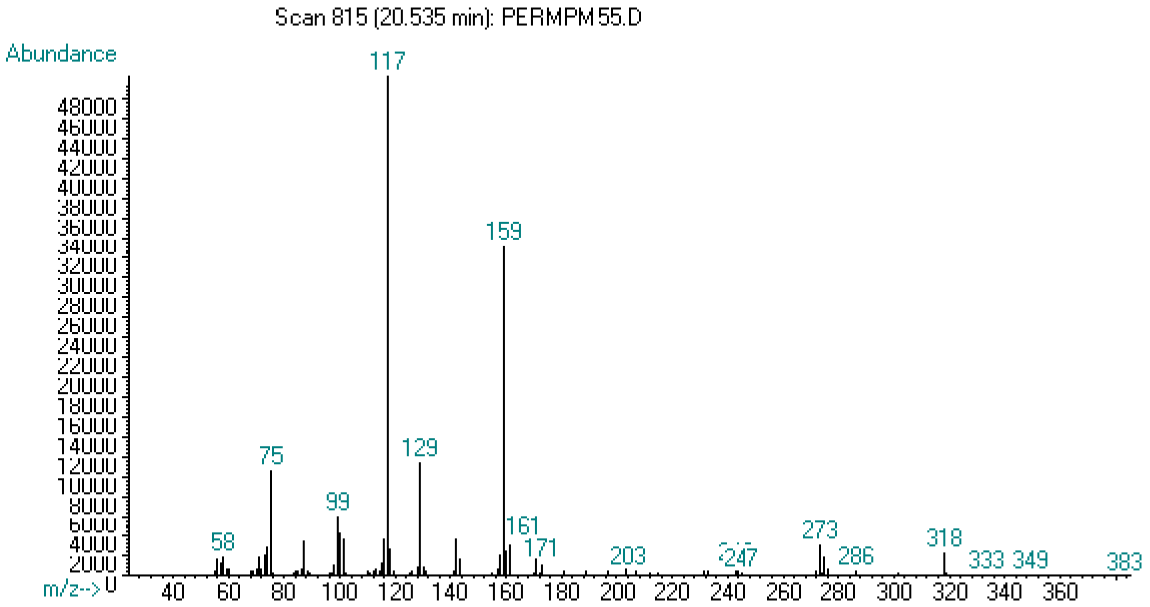
Scan 815 (20.535 min): PERMPM55.D
Abundance
m/z-->
117
159
129
75
99
58
161
171
203
247
273
286
318
333 349
383

D

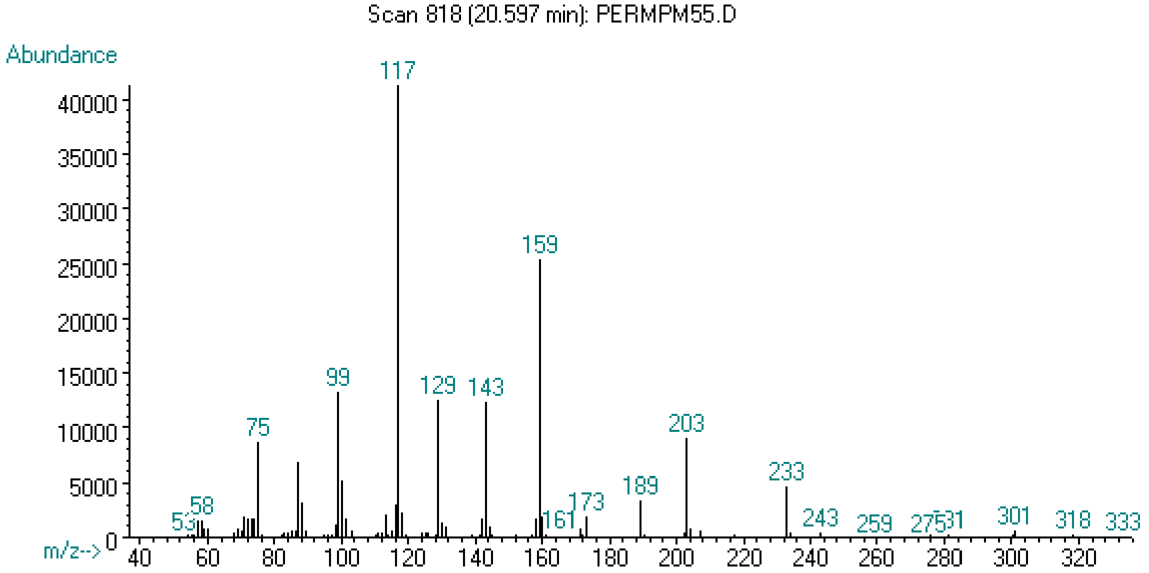
Scan 818 (20.597 min): PERMPM55.D
Abundance
m/z-->
117
159
99
129
143
75
203
233
189
173
161
53
58
243
259
301
318
333
