# Supplementary material for: Antigenic and Structural Properties of the Lipopolysaccharide of the Uropathogenic Proteus mirabilis Dm55 Strain Classified to a New O85 Proteus Serogroup
Source: Int J Mol Sci. 2023 Nov 16;24(22):16424. doi: 10.3390/ijms242216424 (PMC10671486; doi:10.3390/ijms242216424)
Supplement: Supplementary file 1 [file ijms-24-16424-s001.zip › Supplementary Figures S4 and S5.pdf]

Supplementary Materials:

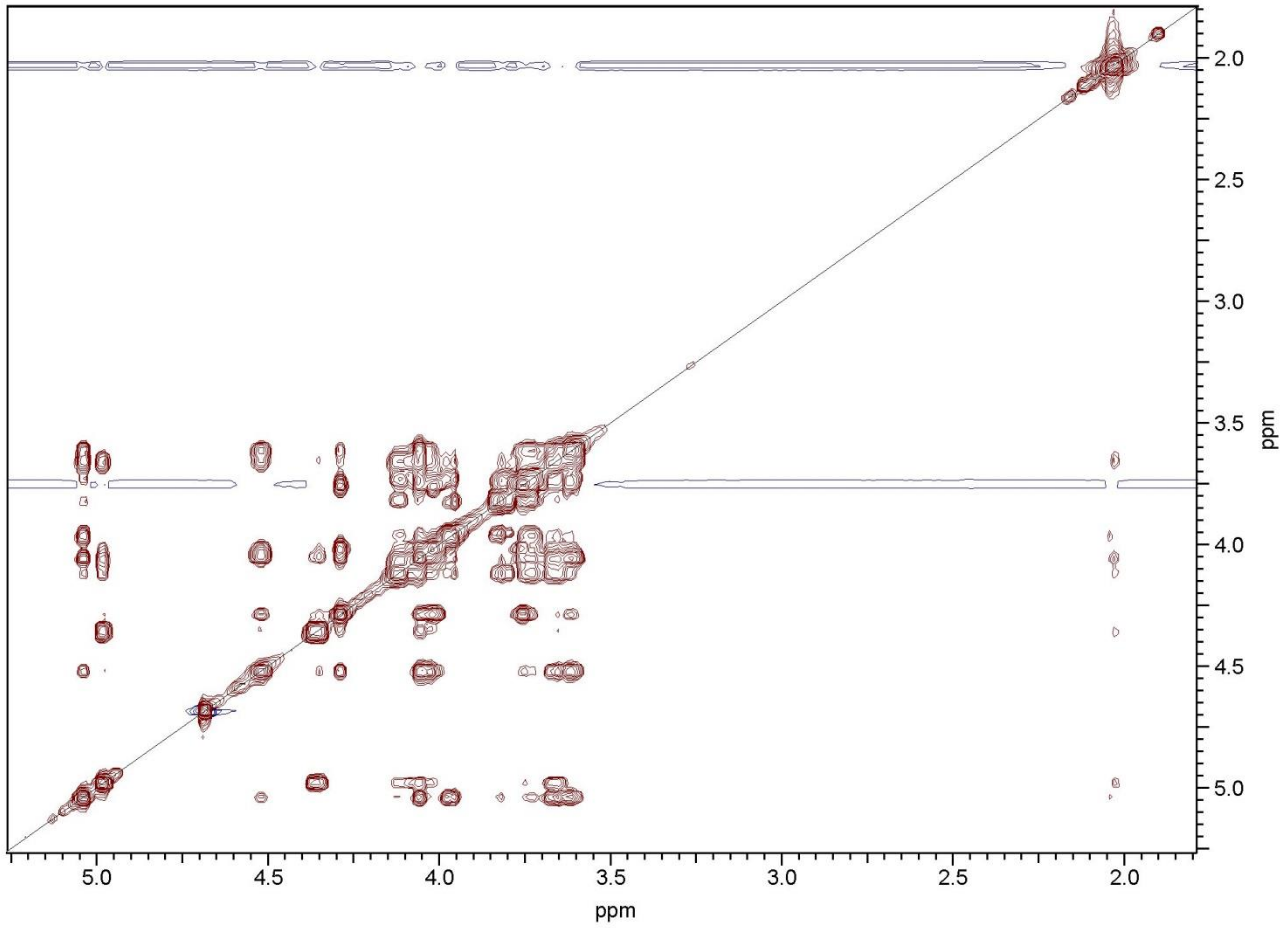

Figure S4. Full range of $^{1}H,^{1}H$ NOESY spectrum of the OPS of *P. mirabilis* strain Dm55.

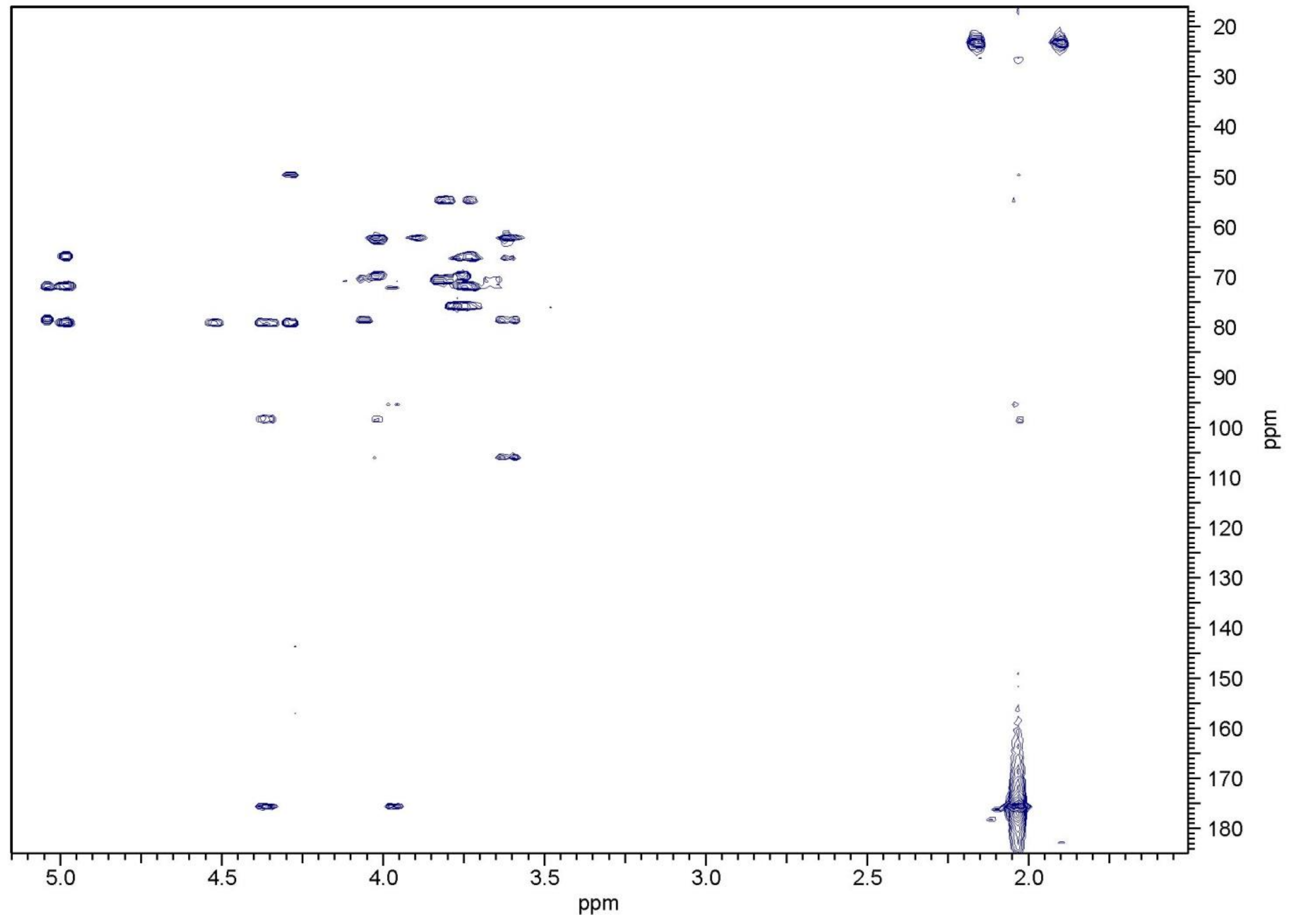

Figure S5. Full range of $^{1}H,^{13}C$ HMBC spectrum of the OPS of *P. mirabilis* strain Dm55.
