# Supplementary material for: Antigenic and Structural Properties of the Lipopolysaccharide of the Uropathogenic Proteus mirabilis Dm55 Strain Classified to a New O85 Proteus Serogroup
Source: Int J Mol Sci. 2023 Nov 16;24(22):16424. doi: 10.3390/ijms242216424 (PMC10671486; doi:10.3390/ijms242216424)
Supplement: Supplementary file 1 [file ijms-24-16424-s001.zip › Supplementary Tables S1 and S2.pdf]

Table S1. The absorbance values in the reactions of the serially diluted Dm55 antiserum with the cross-reacting *Proteus* LPSs.

The absorbance (A_{405}) was measured by using a Multiskan Go microplate reader. Antibody titers were determined as the highest antiserum dilution giving an absorbance of ≥0.2 (marked in yellow).

Proteus spp. LPS	The absorbance (A_{405}) measured for each Dm55 antiserum dilution (x 1000):											
	2	4	8	16	32	64	128	256	512	1024	2048	4096
O54	1.07	1.07	0.607	0.432	0.283	0.252						
O9	2.39	1.97	1.8	1.39	0.775	0.49	0.246					
O13	3.09	3.09	3.1	2.92	2.44	1.59	0.89	0.466	0.211			
O64	0.382	0.335	0.278	0.222	0.221							
O62	0.994	0.816	0.66	0.47	0.399	0.277						
O63	0.592	0.453	0.355	0.251								
O52	0.551	0.403	0.285	0.204								
O59	0.389	0.293	0.238	0.177								
O72	0.524	0.564	0.492	0.479	0.306	0.202						

Table S2. The absorbance values in the reactions of the serially diluted *Proteus* spp. antisera with the homologous LPSs.

The absorbance (A_{405}) was measured by using a Multiskan Go microplate reader. Antibody titers were determined as the highest antiserum dilution giving an absorbance of ≥0.2 (marked in yellow).

Proteus spp. LPS	The absorbance (A_{405}) measured for each dilution (x 1000) of the antisera homologous to the tested LPSs:											
	2	4	8	16	32	64	128	256	512	1024	2048	4096
Dm55	3.14	3.09	3.09	3.11	3.09	3.07	2.91	2.26	1.46	0.672	0.359	0.18
O54	3.1	3.1	3	2.6	1.81	1.14	0.598	0.295				
O9	3.01	2	0.947	0.655	0.353	0.361	0.323	0.288				
O13	3.08	3.06	3.07	2.93	2.61	1.7	1.12	0.599	0.292			
O64	3.1	3.09	3.09	3.08	2.99	2.72	1.89	1.27	0.64	0.318	0.218	
O62	3.08	3.07	3.06	2.91	2.73	1.9	1.37	0.808	0.65	0.399	0.365	0.335
O63	3.1	3.06	3.06	2.9	2.46	1.62	0.908	0.525	0.276			
O52	3.1	3.1	3.07	2.95	2.95	2.77	1.74	1.46	0.872	0.431	0.186	
O59	3.05	3.02	2.66	1.9	1.12	0.539	0.311					
O72	3	2.75	2.28	1.58	0.72	0.417	0.187					
